# Effects of low-dose furosemide combined with aminophylline on the renal function in septic shock patients

**DOI:** 10.1080/0886022X.2023.2185084

**Published:** 2023-03-01

**Authors:** Zhenhua Mai, Yaying Tan, Yang Zhu, Zilong Yang, Hongpeng Chen, Shuting Cai, Wangwang Hu, Xiaoyan Wang, Fenghua Ding, Liehua Deng

**Affiliations:** a Department of Critical Care Medicine, Affiliated Hospital of Guangdong Medical University, Zhanjiang, Guangdong, China; b Zhanjiang Key Laboratory of Organ Injury and Protection and Translational Medicine, Guangdong, China

**Keywords:** Septic shock, aminophylline, furosemide, acute kidney injury, mortality

## Abstract

**Background:**

To investigate the effects of low-dose furosemide and aminophylline on the renal function in patients with septic shock.

**Methods and Results:**

A total of 109 eligible septic shock patients in the intensive care unit were randomly divided into a control group (*n* = 55) and an intervention group (*n* = 54). The control group received normal saline, and the intervention group received low-dose furosemide (0.048 mg/kg.h^−1^) with aminophylline (0.3 mg/kg.h^−1^). The primary outcomes included the levels of serum creatinine (Scr), creatinine clearance rate (Ccr), blood urea nitrogen (BUN), glomerular filtration rate (GFR), and urine output on admission and on days 3, 7 and 14. The secondary outcomes were the sequential organ failure assessment (SOFA) scores, continuous renal replacement therapy (CRRT) time and intensive care unit (ICU) mortality, hospital mortality and 28-day mortality. There were no significant differences in the levels of Scr, Ccr, BUN, or GFR between the two groups, while the urine output was higher in the intervention group on days 3, 7, and 14. Compared with the control group, the SOFA scores, ICU mortality, hospital mortality and 28-day mortality were significantly lower in the intervention group on days 3, 7, and 14, the CRRT time was shorter, and the cumulative fluid balance was lower on days 3 and 7 in the intervention group.

**Conclusions:**

Although low-dose furosemide and aminophylline have fewer protective effects on the renal function in septic shock patients, they could reduce the CRRT time and improve the prognosis.

## Introduction

Acute kidney injury (AKI) is common in intensive care units (ICUs), and it has a 41.3% morbidity and a 25.7% mortality [1,2]. The major reasons for AKI include septic shock, hypovolemia, and the use of drugs that are renal toxic in critically ill patients [1,3], and its mechanism might be associated with an inner perfusion imbalance secondary to a severe infection and septic shock [4].

AKI is one of the major complications of septic shock [5,6]. The morbidity of AKI is 35.8% for patients in the ICU [7], and 50% of AKI cases are caused by severe infection and septic shock [8]. The morbidity of AKI was shown to be 42.1% in 33,375 patients with systemic infection, and it reached 70% in the patients with severe infections [9]. In brief, the high morbidity and mortality in septic shock patients who are complicated with AKI prompted us to find a more effective way to prevent it.

Furosemide can increase the prostaglandin E2 (PGE_2_) levels by inhibiting prostaglandin activity, leading to blood vessel dilatation, an increase in the renal blood flow (especially deep renal cortex) and an improvement in the glomerular filtration rate (GFR). It also reduces the renal tubular oxygen demand by impairing tubular Na^+^ reabsorption [10] and attenuates the AKI-induced kidney metabolic demand and oxidative stress [10–13].

Relevant studies have reported that aminophylline can increase renal blood flow, which has potential benefits in the prevention and treatment of AKI [14–16]. In the renal vascular system, adenosine can produce vasoconstriction. As an adenosine receptor antagonist, aminophylline can block the vasoconstriction of adenosine in the kidney, which leads to diuresis [17].

Recent studies have shown that furosemide alone fails to improve the renal function in patients with septic shock [18], but the effect of low-dose furosemide and aminophylline on the renal function in septic shock patients remains unclear.

## Materials and methods

This is a randomized, single-blind, placebo-controlled trial. Between January 2017 and December 2018, 325 septic shock patients were recruited from the ICU of the Affiliated Hospital of Guangdong Medical University. Septic shock was diagnosed according to the 2016 international guidelines of severe sepsis and septic shock 2016 [19]. The patients who had successful fluid resuscitation were included. The exclusion criteria included patients: aged <18 y or >80 y, who were pregnant; with a kidney transplant history, renal artery stenosis, unilateral renal agenesis, chronic renal failure (serum creatinine >2 mg/dL, chronic dialysis or creatinine clearance rate <30 mL/min), an allergy to furosemide or aminophylline, glomerulonephritis-induced acute renal failure (ARF), whose resuscitation had failed; or who were unwilling to accept the continuous renal replacement therapy (CRRT) treatment.

**Figure 1.**
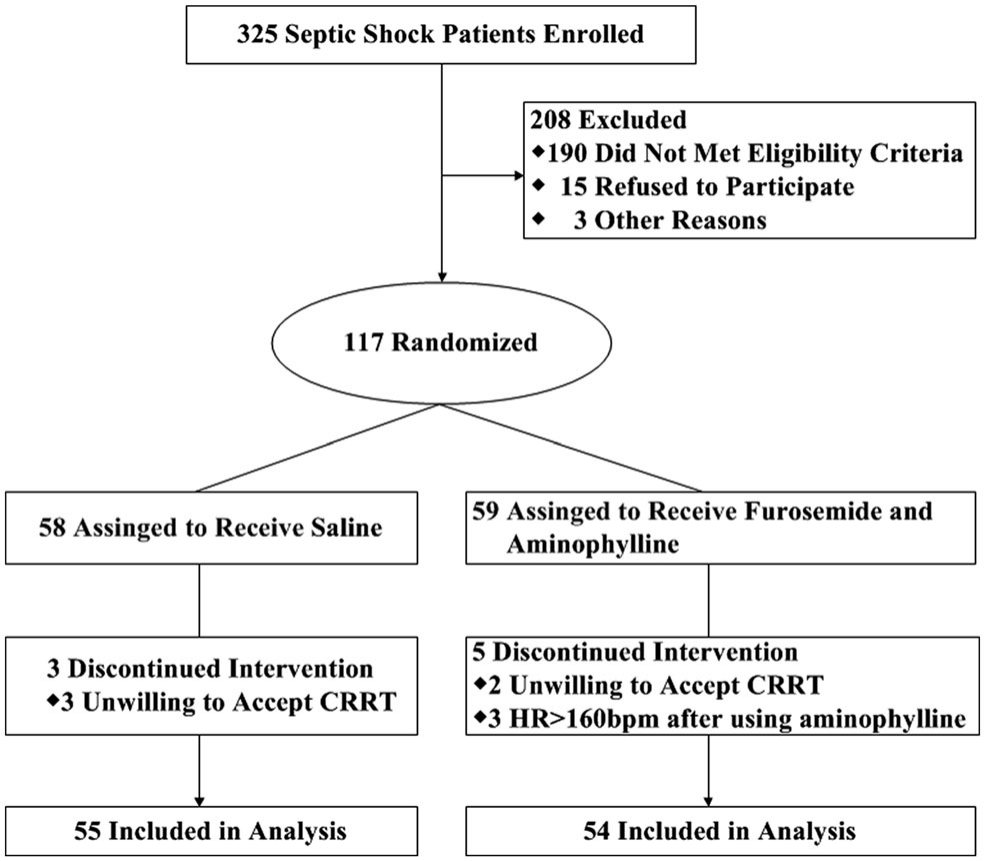
Flowchart of trial patients. HR: heart rate; CRRT: continuous renal replacement therapy.

This study was reviewed and was approved by the Medical Ethical Committee of the Affiliated Hospital of Guangdong Medical University (2016-093KT); The Medical Ethics Committee approves the requirement for informed consent; and the study was conducted in accordance with the Helsinki Declaration. Written informed consent was obtained from the patients or the patients’ legally authorized representatives. The patients were randomized 1:1 to receive furosemide and aminophylline or placebo (Figure 1) using a randomization generated by the VCU Health Investigational Drug Service by means of the Research Randomizer at http://www.randomizer.org. The study was registered at chictr.org.cn (chiCTR2000040822).

A total of 109 patients were randomly divided into a control group (*n* = 55) and an intervention group (*n* = 54) (Figure 1). All patients received septic shock bundle treatment for 6 h, such as fluid resuscitation, vasopressors, antibiotics and so on. The intervention group was treated with low-dose furosemide (0.048 mg/kg.h^−1^) and aminophylline (0.3 mg/kg.h^−1^) for 7 days. This dose of aminophylline can maintain the blood drug concentration at 10-20 µg/ml degrees according to the drug use instructions to ensure the best treatment effect and avoid adverse reactions. The administration of normal saline in the control group was conducted with a total dose of 48 mL and an average rate of 2 mL/h. The duration of normal saline administration in the control group was also 7 days. According to the 2012 Kidney Disease: Improving Global Outcomes (KDIGO) guidelines, CRRT is initiated when AKI grade 2 is combined with volume overload or hyperkalemia or metabolic acidosis. The serum creatinine (Scr), blood urea nitrogen (BUN), GFR, creatinine clearance rate (Ccr), urine output, hematocrit, albumin, mean arterial pressure (MAP), central venous pressure (CVP), acute physiology and chronic health evaluation II (APACHE II) score, sequential organ failure assessment (SOFA), and CRRT time were recorded daily.

The primary outcomes were the Scr, BUN, GFR, Ccr, and urine output on days 3, 7, and 14. The secondary outcomes were the APACHE II score, SOFA, MAP, CVP, hematocrit and albumin on days 3, 7, and 14, as well as the CRRT time, ICU mortality, hospital mortality and 28-day mortality.

SPSS 23.0 software is used for data analysis. The primary outcomes (Scr, BUN, GFR, Ccr, and urine output on days 3, 7, and 14.) were data with normal distribution, and *t*-test was used to compare the statistical differences between the two groups. In the secondary outcomes, data conforming to the normal distribution (APACHE II score, SOFA, MAP, CVP, hematocrit, albumin, Time of CRRT) were analyzed by *t*-test, while ICU mortality, hospital mortality and 28-day mortality were compared by chi square test. The survival rate was analyzed by the log-rank test and a Kaplan–Meier curve. *p* < 0.05 was considered significant.

## Results

The baseline demographics of the 109 eligible septic shock patients are shown in Table 1. There were no significant differences in age, sex, APACHE II score, SOFA score, MAP, CVP, hematocrit, albumin, Scr, BUN, GFR, Ccr, urine output, or AKI incidence between the two groups on admission (Table 1).

**Table 1.** Baseline characteristics of all patients ($\bar{x}$±*s*).

Variable	Control group(*n* = 55)	Intervention group (*n* = 54)	*p*-Value
Demographic data, No. (%)			
Age (years)	68±13	69±9	0.290
Male/female	18/8	14/8	0.682
Incidence of AKI (*n*, %)	29(52.7)	26(48.1)	0.633
Weight (kg),	56.6 ± 6.0	54.6±7.7	0.129
APACHE II score	18.3 ± 5.2	17.5±6.4	0.442
SOFA score	12.5±5.6	11.3±4.1	0.178
MAP (mmHg)	70±13	73±16	0.332
CVP (cmH_2_0)	6.7±3.4	7.7±2.4	0.077
GFR (ml/min)	51.7±34.8	55.9±30.6	0.425
Ccr (ml/min)	41.7±23.0	44.8±28.3	0.539
Scr (umol/l)	151.6±87.6	123.0±64.7	0.055
Urine output [ml/(kg·h)]	1.4±1.2	1.3±0.6	0.910
BUN (mmol/l)	17.4±15.6	12.5±5.6	0.087
Hematocrit (%)	30.1±5.2	27.5±9.2	0.072
Albumin (g/l)	29.6±4.9	29.7±6.7	0.930
Septic shock etiology, No. (%)			
Thorax	25(45.4)	28(51.8)	0.392
Abdomen	19(34.5)	17(31.5)	0.892
Urinary tract	7(12.7)	5(9.3)	0.786
Blood stream	3(5.5)	2(3.7)	0.983
unknown	1(1.8)	2(3.7)	0.237
Admission source, No. (%)			
Emergency department	17(30.9)	15(27.8)	0.882
Outside hospital transfer	10(18.2)	9(16.7)	0.965
Inpatient ward transfer	22(40)	23(42.6)	0.640
Operating room	2(3.6)	3(5.6)	0.351
Direct admission	4(7.3)	4(7.4)	0.694
Comorbidities, NO. (%)			
Hypertension	17(30.9)	15(27.3)	0.882
Diabetes mellitus	24(43.6)	28(50.9)	0.295
COPD	13(23.6)	11(20)	0.857
Chronic heart failure	8(14.5)	6(10.9)	0.803

APACHE II: Acute Physiology and Chronic Health Evaluation II scores; SOFA: Sequential Organ Failure Assessment scores; MAP: mean artery pressure; CVP: central venous pressure; GFR: glomerular filtration rate; Ccr: creatinine clearance rate; Scr: creatinine levels; BUN: blood urea nitrogen; COPD: chronic obstructive pulmonary diseases.

The primary outcomes showed that there was no statistically significant difference in the Scr, BUN, GFR, or Ccr between the intervention group and the control group on admission and on days 3, 7, and 14. The urine output was higher in the intervention group on days 3, 7, and 14 (Table 2).

**Table 2.** Primary outcomes in a trail of low dose furosemide and aminophylline in patients with septic shock.

Variables	Days	Control group (*n*)	Intervention group (*n*)	Difference, coefficient (95% CI)	*p*-Value
GFR (ml/min)	3	52.2±37.0(53)	57.8±31.6(53)	10.0 (−25.8 − 14.6)	0.403
	7	48.9±38.4(49)	54.2±36.8(50)	11.2 (−27.9 − 17.3)	0.485
	14	51.7±34.8(42)	52.1±35.4(48)	10.6 (−21.7 − 30.0)	0.960
Ccr (ml/min)	3	48.1±31.5(53)	44.8±28.0(53)	9.2 (−19.6 − 17.4)	0.573
	7	46.5±50.7(49)	42.8±31.2(50)	12.6 (−21.7 − 29.2)	0.659
	14	47.4±40.1(42)	41.4±30.3(48)	10.7 (−15.7 − 27.6)	0.425
Scr (umol/l)	3	143.9±93.4(53)	128.2±74.3(53)	43.0 (1.0 − 176.2)	0.340
	7	193.6±133.9(49)	156.6±104.1(50)	35.9 (−35.4 − 109.3)	0.128
	14	159.2±101.5(42)	168.2±117.7(48)	33.1 (−75.9 − 57.8)	0.699
BUN (mmol/l)	3	15.3±12.1(53)	11.9±6.5(53)	2.7 (−1.1 − 10.0)	0.073
	7	19.4±11.7(49)	17.5±14.4(50)	3.9 (−6.0 − 9.8)	0.475
	14	15.3±8.7(42)	16.4±14.7(48)	3.6 (−8.6 − 6.3)	0.640
Urine output [ml/(kg·h)]	3	1.3±1.0(53)	1.7±0.7(53)	0.3 (−0.9 − 0.1)	0.024
	7	1.5±1.3(49)	2.4±1.7(50)	0.3 (−0.9 − 0.4)	0.009
	14	1.7±1.0(42)	2.5±1.1(48)	0.3 (−1.3 − 0)	<0.001

GFR: glomerular filtration rate; Ccr: creatinine clearance rate; Scr: serum creatinine levels; BUN: blood urea nitrogen.

**Figure 2.**
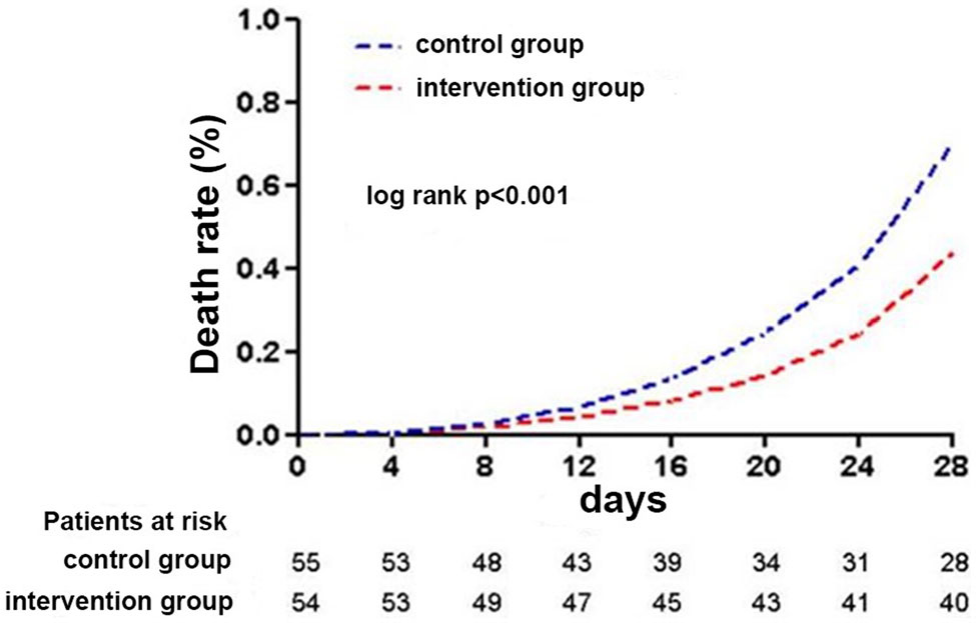
Comparison of 28-day mortality between the control group and the intervention group.

The secondary outcomes showed that the APACHE II score, MAP, hematocrit and albumin were not significantly different between the intervention group and the control group on admission and on days 3, 7, and 14, while CVP on days 7 and 14, SOFA, CRRT time, ICU mortality, hospital mortality and 28-day mortality were significantly lower in the intervention group than in the control group (Table 3, Figure 2).

**Table 3.** Secondary outcomes in a trail of low dose furosemide and aminophylline in patients with septic shock.

Variables	Days	Control group (*n* or %)	Intervention group (*n* or %)	Difference, coefficient (95% CI*)*	*p*-Value
APACHE II score	3	17.4±4.7(53)	16.1±5.24(53)	1.4 (−1.5 − 4.3)	0.158
	7	18.3±5.5(49)	16.4±8.2(50)	2.1 (−2.3 − 6.1)	0.179
	14	17.8±6.9(42)	14.9±9.3(48)	2.5 (−2.0 − 7.9)	0.092
SOFA score	3	12.1±5.6(53)	8.4±3.2(53)	1.3 (1.1 − 6.3)	<0.001
	7	11.9±5.8(49)	7.6±2.8(50)	1.4 (1.6 − 7.1)	<0.001
	14	12.1±7.8(42)	7.1±6.6(48)	2.4 (−0.8 − 8.8)	0.001
MAP (mmHg)	3	80.3±13.1(53)	79.4±5.7(53)	2.9 (−4.9 − 6.7)	0.663
	7	78.7±13.8(49)	79.4±13.3(50)	4.0 (−8.8 − 7.5)	0.820
	14	72.1±19.6(42)	72.5±22.2(48)	6.3 (−13.1 − 12.4)	0.945
CVP (cmH_2_O)	3	7.7±3.9(53)	8.8±3.0(53)	1.0 (−3.2 − 0.8)	0.077
	7	9.3±2.7(49)	7.8±3.20(50)	0.9 (−4.3 − 0.6)	0.013
	14	9.2±2.742)	7.2±3.0(48)	1.0 (−3.5 − 0.7)	0.002
Hematocrit (%)	3	27.4±5.3(53)	26.1±7.2(53)	1.9 (−2.5 − 5.0)	0.315
	7	27.5±4.6(49)	26.7±4.0(50)	1.3 (−1.7 − 3.4)	0.326
	14	27.2±3.9(42)	26.9±5.6(48)	1.5 (−2.6 − 3.2)	0.743
Albumin (g/l)	3	31.4±4.9(53)	32.5±7.0(53)	1.8 (−6.8 − 0.4)	0.222
	7	34.5±7.0(49)	35.0±5.4(50)	1.6 (−4.0 − 2.3)	0.748
	14	34.7±5.1(42)	35.6±3.6(48)	1.3 (−4.6 − 0.8)	0.359
Time of CRRT (h)		42.4±47.6	20.1±13.3	17.5 (−2.9 − 67.5)	0.001
ICU mortality		20 (36.4)	9 (16.7)		0.020
Hospital mortality		23(41.8)	11 (20.3)		0.015
28-day mortality		27 (49.1)	14 (25.9)		0.012

APACHE II: Acute Physiology and Chronic Health Evaluation II scores; SOFA: Sequential Organ Failure Assessment scores; MAP: mean artery pressure; CVP: central venous pressure.

The cumulative fluid balance was markedly lower in the intervention group on days 3 and 7, while there was no significant difference on day 1 compared with the control group (Table 4).

**Table 4.** Comparison of cumulative fluid balance on days 1, 3 and 7($\bar{x}$±*s*).

Group	Cumulative fluid balance (ml)		
	Day 1 (*n*)	Day 3 (*n*)	Day 7 (*n*)
Control group	573 ± 2484(55)	246 ± 1550(53)	−1073 ± 1937(49)
Intervention group	−358 ± 3367(54)	−581 ± 536(53)	−4143 ± 3729(50)
Difference, coefficient (95% CI)	667 (−412 − 2274)	325 (166 − 1489)	880 (1295 − 4844)
*p*-Value	0.170	0.016	0.001

Cumulative fluid balance volume (ml): cumulative fluid intake volume-cumulative fluid output volume.

## Discussion

In this randomized placebo-controlled trial, combined furosemide and aminophylline improved the urine output, fluid balance and SOFA score and reduced the ICU, hospital and 28-day mortality, but this treatment had no impact on the BUN, Scr, Ccr, and GRF.

This result is undoubtedly gratifying. In theory, furosemide can improve the renal function and can increase the urine output by increasing the renal blood flow. However, the results of some studies do not support this hypothesis. The use of furosemide does not improve the renal function, although it can increase the urine output [20]. The KDIGO guidelines do not recommend diuretics as a treatment for patients with AKI [21]. A recently published large-sample study [22] showed that furosemide can promote the recovery of the renal function of critically ill patients with AKI and can reduce the hospital mortality, especially in patients who had stage 2–3 AKI UO, and these results were similar to ours.

Usually, we use furosemide because the patient’s urine output is reduced, and our hope is to achieve our treatment goal of a negative fluid balance through the use of diuretics. Our study found that the intervention group had more urine output, a better negative fluid balance, a lower CVP, a reduced CRRT duration, a reduced SOFA score, and significantly reduced ICU mortality, hospital mortality, and 28-day mortality. We also noticed that the 28-day mortality rate in the control group was very high, almost twice as high as in the intervention group. We think this may be because the subjects in this experiment were septic shock patients with high APACHE II scores, low number of cases, and high mortality. In the Dinna N Cruz and Glenn H studies, enrolled patients with septic shock had APACHE II scores and 28-day mortality similar to ours [23,24].We are evaluating whether the intervention group had a better negative fluid balance, which ultimately affects the patient’s prognosis and reduces the mortality rate. The septic shock patients who were enrolled in this group had undergone adequate fluid resuscitation; therefore, the fluid management of critically ill patients is essential. Volume overload is difficult to avoid in critically ill patients, and this type of volume overload increases the death rate of critically ill patients [25]. Strict fluid management can improve the prognosis. An RCT previously showed that in critically ill patients with AKI, restricted fluid management can achieve a better fluid balance, reduce the renal replacement therapy, and reduce the occurrence of adverse events. Unfortunately, of the 997 patients included in the study, only 100 eventually entered the trial for analysis [26].

Some patients have a higher risk of hyperchloremia after undergoing full fluid resuscitation, especially after using normal saline. Results from a large study [27] showed that compared with balanced crystalloid solution, the saline group had a higher incidence of major adverse renal events (death from any cause, new renal replacement therapy, persistent renal insufficiency). This indicates that there might be an unexplored association between hyperchloremia and the above outcomes. *Post hoc* analysis of the ‘HYPER2S’ trial [28] suggested that acidosis caused by hyperchloremia was more frequent, but there was no clear evidence demonstrating the connection of hyperchloremia with an increased risk for AKI or mortality.

Our research has certain limitations. It should be pointed out that some patients may have diuretic resistance, especially those who took diuretic drugs for a long time. Similarly, we did not have statistics and analysis of norepinephrine and nephrotoxic drugs. We cannot be sure that the improvement in the prognosis and the reduction in the mortality are the direct result of the combined use of furosemide and aminophylline. The same results were found in a study using furosemide alone, although that study was a retrospective study [22]. In addition, the treatment strategies, including mechanical ventilation, blood pressure drugs, and nutrition, of critically ill patients are directly related to the prognosis. According to the 2012 KDIGO guidelines [21], urine volume and creatinine are used to grade AKI. In this study, BUN and Scr were only selected to evaluate renal function. We will add indicators to evaluate renal function in subsequent large-sample studies. Finally, the advantage of this study is that it is forward-looking, but it is also a single center with a small sample size. Therefore, a larger sample sized study is needed to verify our results.

## Conclusion

In summary, the use of a low-dose combination of furosemide and aminophylline may improve the prognosis of patients potentially through a negative fluid balance, but it has no effect on the recovery of the renal function indexes in patients with septic shock.

## Data Availability

All data generated or analyzed during this study are included in this article and its supplementary information files.
